# Metabolomics and Cell Therapy in Diabetes Mellitus

**DOI:** 10.22088/IJMCM.BUMS.8.2.41

**Published:** 2019-05-30

**Authors:** Bagher Larijani, Parisa Goodarzi, Moloud Payab, Sepideh Alavi-Moghadam, Fakher Rahim, Nikoo Bana, Mina Abedi, Maryam Arabi4, Hossein Adibi, Kambiz Gilany, Babak Arjmand

**Affiliations:** 1 *Endocrinology and Metabolism Research Center, Endocrinology and Metabolism Clinical Sciences Institute, Tehran University of Medical sciences, Tehran, Iran.*; 2 *Brain and Spinal Cord Injury Research Center, Neuroscience Institute, Tehran University of Medical Sciences, Tehran, Iran.*; 3 *Obesity and Eating Habits Research Center, Endocrinology and Metabolism Molecular-Cellular Sciences Institute, Tehran University of Medical Sciences, Tehran, Iran.*; 4 *Cell Therapy and Regenerative Medicine Research Center, Endocrinology and Metabolism Molecular-Cellular Sciences Institute, Tehran University of Medical Sciences, Tehran, Iran.*; 5 *Health Research Institute, Thalassemia and Hemoglobinopathies Research Center, Ahvaz Jundishapur University of Medical Sciences, Ahvaz, Iran.*; 6 *Metabolomics and Genomics Research Center, Endocrinology and Metabolism Molecular-Cellular Sciences Institute, Tehran University of Medical Sciences, Tehran, Iran.*; 7 *Diabetes Research Center, Endocrinology and Metabolism Clinical Sciences Institute, Tehran University of Medical Sciences, Tehran, Iran.*; 8 *Department of Biomedical Sciences, University of Antwerp, Belgium.*; 9 *Integrative Oncology Department, Breast Cancer Research Center, Motamed Cancer Institute, ACECR, Tehran, Iran.*

**Keywords:** Cell-based therapy, diabetes mellitus, metabolic diseases, metabolomics, metabolic pathways

## Abstract

Diabetes with a broad spectrum of complications has become a global epidemic metabolic disorder. Till now, several pharmaceutical and non-pharmaceutical therapeutic approaches were applied for its treatment. Cell-based therapies have become promising methods for diabetes treatment. Better understanding of diabetes pathogenesis and identification of its specific biomarkers along with evaluation of different treatments efficacy, can be possible by clarification of specific metabolic modifications during the diabetes progression. Subsequently, metabolomics technology can support this goal as an effective tool. The present review tried to show how metabolomics quantifications can be useful for diabetic monitoring before and after cell therapy. Cell therapy is an alternative approach to achieve diabetes treatments goals including insulin resistance amelioration, insulin independence reparation, and control of glycemia. OMICs approaches provide a comprehensive insight into the molecular mechanisms of cells features and functional mechanism of their genomics, transcriptomics, proteomics, and metabolomics profile which can be useful for their therapeutic application. As a modern technology for the detection and analysis of metabolites in biological samples, metabolomica can identify many of the metabolic and molecular pathways associated with diabetes and its following complications.

Metabolism is the sum of chemical reactions which break complex organic molecules down to obtain energy. Complex modifications in glucose and lipid metabolism can cause metabolic diseases such as diabetes mellitus (DM). DM as a global epidemic disorder is responsible for 4 million deaths annually (1, 2). Two types of DM are type1 (T1DM) and type2 (T2DM) (3, 4). Both DM types have a broad range of conventional treatments, which have serious cons points along with their pros points (5). More recently, by successful development in the therapeutic application of stem cells, the use of stem cells to improve diabetic patientswas remarkable as a novel alternative method (6). Indeed, stem cell therapy can improve diabetes treatment through differentiation of stem cells into insulin- producing cells, regeneration of pancreas, amending of insulin resistance, and modulation of immune system (7, 8). Due to extensive metabolic rewiring of stem cells during differentiation, reprogramming, and proliferation processes, evaluation of stem cell metabolism via metabolomics approaches have a significant role in controlling stem cell fates (9, 10). On the other hand, metabolomics can distinguish different stem cell types based on their molecular and metabolite biomarkers (11). Additionally, metabolomics is a potent tool to analyze the complex pathways of disease progression over time, and also it can assay the effects of various treatments (12, 13). Accordingly, the aim of this article was to introduce metabolomics application in cell therapy and regenerative medicine.

## Diabetes as a metabolic disorder

Metabolic disorders occur in specific genetic and chemical conditions in which normal cell metabolism is impaired. According to international diabetes federation report, over 425 million people were affected by DM as an example of metabolic disorders around the world (14). DM diagnosis can be confirmed by hyperglycemia (fasting hypergly-cemia with blood sugar higher than 130 mg/dl and postprandial hyperglycemia with blood sugar higher than 180 mg/dl) resulted from deficiencies in secretion and action of insulin (15). Involved pathogenic processes in DM are including autoimmune destruction of the pancreatic β-cells followed by insulin deficiency (type 1) and islet cell dysfunction along with insulin resistance (type 2) (13, 16). Some of the long term DM complications including nephropathy leading to renal failure, reduced visual acuity in retinopathy, and peripheral neuropathy with a risk of foot ulcer, introduce it as a global health care burden (16-18). Hereupon, treatment of DM is crucial to decrease its later complication risks.

## Treatment and management of diabetes

In order to manage and control DM, a combination therapy including drugs and lifestyle modification is required. With this in view, American Diabetes Association (ADA) and the European Association of Diabetes (EASD) recommended lifestyle modification as the first step of DM management. A suitable diet, regular physical activity and the ideal amount of sleep time are important factors in development of healthier lifestyle. Although a healthy lifestyle is preferable, following a special lifestyle is exhausting for a long time (19, 20). On the other hand, some pharmacological interventions (Table 1) are required along with a change in lifestyle.

Despite the different types of pharmacological treatments, and application of some alternative methods such as hydrotherapy, acupuncture, and dietary supplements, definitive treatments for DM has not been announced yet. In recent years, cell-based therapies as hopeful approaches to the treatment of chronic disorders such as DM have been progressed from bench-to-bedside (31-36).

**Table 1:** Advantages and disadvantages of diabetes medicines

**Class of Medications**	**Example**	**Advantages**	**Disadvantages**	**Mechanism of action**	**References**
**Biguanidas**	Metformin (Glucophag)	Reducing hepatic glucose, fasting glycemia, Hemoglobin A1C	Weight loss Anorexia Nausea Abdominal discomfort Diarrhea	Reducing hepatic glucose output through inhibition of gluconeogenesis	(19, 21-24)
**Sulfonylures**	Glibenclamie (Daonil), Gliclazide (Glizid(	Secreting insulin Decreasing glycemia, Hemoglobin A1C	Weight gain Hypoglycemia	Increasing insulin secretion regulated by ATP-sensitive potassium channels	(19, 22, 24, 25)
**Thiazolidinediones (TZDs)**	Glitazones, Pioglitazone	Using glucose by increasing the insulin sensitivity in muscle, fat, and liver tissues	Weight gain Fluid retention with peripheral edema, risk for congestive heart failure	Increasing insulin sensitivity by binding to peroxisome proliferator-activated receptors, improving blood glucose levels by preserving pancreatic beta-cell function	(19, 22, 26, 27)
**Insulin**		Decreasing hemogolobin A1C, Effects on triacylglycerol and HDL cholesterol levels	Weight gain Hypoglycemia Interactions with other medications, Cardiovascular disease	Reducing glucose concentration by increasing glucose uptake or reducing glucose production	(22, 28-31)

## Cell Therapy as an alternative treatment for diabetes

The ultimate goals of the DM treatments are including insulin resistance improvement, insulin independence restoration, and control of glycemia (37). Nowadays, development in the differentiation potential of human stem cells into insulin-producing cells (IPCs) as well as stem cells potential for pancreas regeneration and insulin resistance modification suggests a substantial alternative approach to achieve DM treatments goals (8, 9, 38, 39). According to investigations, embryonic stem cells (ESCs), induced pluripotent stem cells (iPSCs), umbilical cord blood stem cells (UCBs), fetal and adult pancreatic ductal cells, hepatic oval cells, and neural progenitor cells have been introduced as potential sources to generate IPCs (40). However, despite the benefit of their application there are some limitations including immune rejection, genetic abnormalities, and less potential of differentiated IPCs to produce enough insulin. To modulate and control differentiated IPCs for producing enough and proper amount of insulin, understanding and assay of genes, proteins or signaling molecules, and metabolites which are playing significant role, is required (41, 42). 

## Application of multi-OMICs approaches in cell therapy

Recognizing stem cells properties such as self-renewal and differentiation at the molecular level is helpful for stem cell therapy. Additionally, the stem cell fate can be controlled by the complicated functional mechanisms of genome, transcriptome, proteome, and metabolome (43). In this context, OMICs approaches provide a holistic view around the molecular mechanisms of stem cells properties and functional mechanism of their genomic, transcriptomic, proteomic, and metabolomic profile. The processes of cell therapy include chain steps such as cells isolation, culture, and stimulation, which are different based on cells variations. These differences may be related to the complicated genome of donor cells. In other word, analyzing of genes expression related to the particular function of cells could be useful for the examination of cell therapy potency (44). In this regard, genomics technology can analyze the whole genome and gene expression in cells. Furthermore, studies on total RNAs (transcriptome) which are transcribed from cells genome can be also effective. On the other hand, during cell therapy, stem cells can produce some biomolecules and proteins in extracellular space that can each affect the cell based-treatment outcomes (45). Proteomics investigations have a pivotal role in evaluating the functional mechanism of proteins products. However, metabolic analyzing of stem cells metabolome (the complete set of small-molecules which resulted from cell metabolism) via metabolomics studies is more effective than proteome, transcriptome and genome analyzing (12, 46, 47).

## Metabolomics applied to diabetes research

**Fig. 1:**
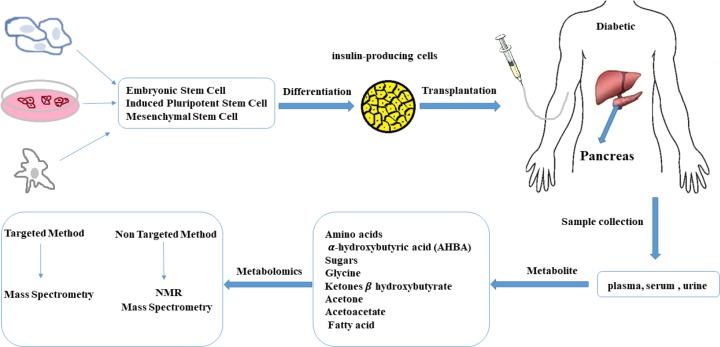
Metabolomics in cell therapy. The level of metabolites can be changed after transplantation. Hence, metabolomics can explain the efficacy of treatment by monitoring the metabolites before and after cell therapy via targeted and non-targeted methods (59).

Metabolomics as a new technology for the detection and measurement of all or a range of metabolites in biological samples can recognize many of the metabolic and molecular pathways (metabolism pathways of fatty acids, amino acids, nucleotides, and etc.) associated with DM and its subsequent complications (12, 48). Indeed, metabolomics can be used to monitor the modification of cells and bio fluids metabolites during the course of the DM in comparison with the normal healthy state (5, 49, 50). Additionally, application of metabolomics methods specifically nuclear magnetic resonance (NMR) and mass spectrometry (MS) approaches to better understand the mechanism of DM related molecular pathways is fruitful for conducting preventive strategies (5, 13, 51, 52). On the other hand, metabolomics can analyze the modification of cellular and bio fluids metabolites in patients before and after application of different therapies e.g. cell therapy (Fig. 1) to realize the efficacy of the selected treatment (53-55).

## Conclusion and future perspectives

Advances in metabolomics approaches have been invaluable for discovering particular disease biomarkers, and to evaluate the mechanisms of action of various novel treatments (i.e. cell-based therapy) as well as understanding the pathogenesis mechanism of diseases (such as diabetes) through targeted or non- targeted methods to improve diagnosis, prediction, and prognosis (56-59). Notable strides in the collection of data and appropriate search of databases for data analyzing have leaded to the rapid development of metabolomics by establishing impressive protocols for sample collection, and data extraction methods along with providing the possibility of computer modeling for disease (metabolomics in systems biology) (60). With the development and progress of metabolomics methods, designing personalized medicines (prescription of particular treatments for each person) in accordance with specific genomic and metabolic profile of each person can be also progressed (61-66). For instance, personalized medicine can be beneficial to manage cases of diabetes with specific strategies that will not be necessarily effective for all patients with same weight, height, and glucose levels. Additionally, it seems that personalized medicine can also be beneficial for diabetes prevention. According to the combination of genetic and metabolic effects on outcomes of therapies, the combination of metabolomics with genomics (such as genome- wide association studies (GWAS)- metabolomics strategy) for decoding the functional mechanisms underlying different treatments can be also helpful. This combination may also clarify which genetic disorders are most simply detectable within the metabolomic investigation (67, 68).

## References

[1] Tabish SA (2007). Is Diabetes Becoming the Biggest Epidemic of the Twenty-first Century?. Int J Health Sci (Qassim).

[2] Larijani B, Ghahari A, Warnock GL (2015). Human fetal skin fibroblasts: Extremely potent and allogenic candidates for treatment of diabetic wounds. Med Hypotheses.

[3] Sas KM, Karnovsky A, Michailidis G (2015). Metabolomics and diabetes: analytical and computational approaches. Diabetes.

[4] American Diabetes A (2009). Diagnosis and classification of diabetes mellitus. Diabetes Care.

[5] Rahim F, Arjmand B, Shirbandi K (2018). Stem cell therapy for patients with diabetes: a systematic review and meta-analysis of metabolomics-based risks and benefits. Stem Cell Investig.

[6] Lee KO, Gan SU, Calne RY (2012). Stem cell therapy for diabetes. Indian J Endocrinol Metab.

[7] Liew CG, Andrews PW (2008). Stem cell therapy to treat diabetes mellitus. Rev Diabet Stud.

[8] Guo T, Hebrok M (2009). Stem cells to pancreatic beta-cells: new sources for diabetes cell therapy. Endocr Rev.

[9] Bhute VJ, Bao X, Palecek SP (2017). Advances in Applications of Metabolomics in Pluripotent Stem Cell Research. Curr Opin Chem Eng.

[10] Shyh-Chang N, Daley GQ, Cantley LC (2013). Stem cell metabolism in tissue development and aging. Development.

[11] Panopoulos AD, Yanes O, Ruiz S (2012). The metabolome of induced pluripotent stem cells reveals metabolic changes occurring in somatic cell reprogramming. Cell Res.

[12] Preet A, Karve TM, Rizk N (2012). Metabolomics: approaches and applications to diabetes research. J Diabetes Metab.

[13] Johnson CH, Ivanisevic J, Siuzdak G (2016). Metabolomics: beyond biomarkers and towards mechanisms. Nat Rev Mol Cell Biol.

[14] Ahmadieh H, Itani H, Itani S (2018). Diabetes and depression in Lebanon and association with glycemic control: a cross-sectional study. Diabetes Metab Syndr Obes.

[15] American Diabetes A (2010). Diagnosis and classification of diabetes mellitus. Diabetes Care.

[16] Siddiqui AA, Siddiqui SA, Ahmad S (2013). Diabetes: Mechanism, pathophysiology and management-A Review. Int J Drug Dev Res.

[17] Beckman JA, Creager MA (2016). Vascular Complications of Diabetes. Circ Res.

[18] Migdalis I, Czupryniak L, Lalic N (2018). Diabetic Microvascular Complications. Int J Endocrinol.

[19] Marin-Penalver JJ, Martin-Timon I, Sevillano-Collantes C (2016). Update on the treatment of type 2 diabetes mellitus. World J Diabetes.

[20] Bagnasco A, Di Giacomo P, Da Rin Della Mora R (2014). Factors influencing self-management in patients with type 2 diabetes: a quantitative systematic review protocol. J Adv Nurs.

[21] DeFronzo RA, Goodman AM (1995). Efficacy of metformin in patients with non-insulin-dependent diabetes mellitus The Multicenter Metformin Study Group. N Engl J Med.

[22] Nathan DM, Buse JB, Davidson MB (2009). Medical management of hyperglycemia in type 2 diabetes: a consensus algorithm for the initiation and adjustment of therapy: a consensus statement of the American Diabetes Association and the European Association for the Study of Diabetes. Diabetes Care.

[23] Gross JL, Kramer CK, Leitao CB (2011). Effect of antihyperglycemic agents added to metformin and a sulfonylurea on glycemic control and weight gain in type 2 diabetes: a network meta-analysis. Ann Intern Med.

[24] Zhao Y, Xu G, Wu W (2015). Type 2 diabetes mellitus-disease, diagnosis and treatment. J Diabetes Metab.

[25] Pimouguet C, Le Goff M, Thiebaut R (2011). Effectiveness of disease-management programs for improving diabetes care: a meta-analysis. CMAJ.

[26] Home PD, Pocock SJ, Beck-Nielsen H (2007). Rosiglitazone evaluated for cardiovascular outcomes--an interim analysis. N Engl J Med.

[27] Singh S, Loke YK, Furberg CD (2007). Thiazolidinediones and heart failure: a teleo-analysis. Diabetes Care.

[28] Lebovitz HE (2011). Insulin: potential negative consequences of early routine use in patients with type 2 diabetes. Diabetes Care.

[29] Donner T, Sarkar S, Feingold KR, Anawalt B, Boyce A (2000). Insulin - Pharmacology, Therapeutic Regimens, and Principles of Intensive Insulin Therapy. Endotext.

[30] Brown PM, Tompkins CV, Juul S (1978). Mechanism of action of insulin in diabetic patients: a dose-related effect on glucose production and utilisation. Br Med J.

[31] Soleimani M, Aghayan HR, Goodarzi P (2016). Stem Cell Therapy-Approach for Multiple Sclerosis Treatment. Arch Neurol.

[32] Pandey A, Tripathi P, Pandey R (2011). Alternative therapies useful in the management of diabetes: A systematic review. J Pharm Bioallied Sci.

[33] Goodarzi P, Aghayan HR, Larijani B (2015). Stem cell-based approach for the treatment of Parkinson's disease. Med J Islam Repub Iran.

[34] Aghayan HR, Arjmand B, Yaghoubi M (2014). Clinical outcome of autologous mononuclear cells transplantation for spinal cord injury: a systematic review and meta-analysis. Med J Islam Repub Iran.

[35] Aghayan HR, Goodarzi P, Arjmand B (2015). GMP-compliant human adipose tissue-derived mesenchymal stem cells for cellular therapy. Methods Mol Biol.

[36] Rahim S, Rahim F, Shirbandi K (2018). Sports Injuries: Diagnosis, Prevention, Stem Cell Therapy, and Medical Sport Strategy. Adv Exp Med Biol.

[37] Sneddon JB, Tang Q, Stock P (2018). Stem Cell Therapies for Treating Diabetes: Progress and Remaining Challenges. Cell Stem Cell.

[38] Scuteri A, Monfrini M (2018). Mesenchymal Stem Cells as New Therapeutic Approach for Diabetes and Pancreatic Disorders. Int J Mol Sci.

[39] Chhabra P, Brayman KL (2013). Stem cell therapy to cure type 1 diabetes: from hype to hope. Stem Cells Transl Med.

[40] Ilgun H, Kim JW, Luo L (2015). Adult Stem Cells and Diabetes Therapy. J Stem Cell Res Transplant.

[41] Warren L, Manos PD, Ahfeldt T (2010). Highly efficient reprogramming to pluripotency and directed differentiation of human cells with synthetic modified mRNA. Cell Stem Cell.

[42] Bruin JE, Saber N, Braun N (2015). Treating diet-induced diabetes and obesity with human embryonic stem cell-derived pancreatic progenitor cells and antidiabetic drugs. Stem Cell Reports.

[43] Nguyen PK, Nag D, Wu JC (2010). Methods to assess stem cell lineage, fate and function. Adv Drug Deliv Rev.

[44] Stroncek DF, Jin P, Wang E (2007). Potency analysis of cellular therapies: the emerging role of molecular assays. J Transl Med.

[45] Maguire G, Friedman P (2013). The systems biology of stem cell released molecules—based therapeutics. ISRN Stem Cells.

[46] Ma H, Sorokin A, Mazein A (2007). The Edinburgh human metabolic network reconstruction and its functional analysis. Mol Syst Biol.

[47] Duarte NC, Becker SA, Jamshidi N (2007). Global reconstruction of the human metabolic network based on genomic and bibliomic data. Proc Natl Acad Sci U S A.

[48] Bain JR, Stevens RD, Wenner BR (2009). Metabolomics applied to diabetes research: moving from information to knowledge. Diabetes.

[49] Pallares-Mendez R, Aguilar-Salinas CA, Cruz-Bautista I (2016). Metabolomics in diabetes, a review. Ann Med.

[50] Guasch-Ferre M, Hruby A, Toledo E (2016). Metabolomics in Prediabetes and Diabetes: A Systematic Review and Meta-analysis. Diabetes Care.

[51] Gonzalez-Franquesa A, Burkart AM, Isganaitis E (2016). What Have Metabolomics Approaches Taught Us About Type 2 Diabetes?. Curr Diab Rep.

[52] Huynh J, Xiong G, Bentley-Lewis R (2014). A systematic review of metabolite profiling in gestational diabetes mellitus. Diabetologia.

[53] Armitage EG, Southam AD (2016). Monitoring cancer prognosis, diagnosis and treatment efficacy using metabolomics and lipidomics. Metabolomics.

[54] Cambiaghi A, Pinto BB, Brunelli L (2017). Characterization of a metabolomic profile associated with responsiveness to therapy in the acute phase of septic shock. Sci Rep.

[55] García- Cañaveras JC, Castell J, Donato MT (2016). A metabolomics cell-based approach for anticipating and investigating drug-induced liver injury. Scientific Reports.

[56] Klein MS, Shearer J (2016). Metabolomics and type 2 diabetes: translating basic research into clinical application. J Diabetes Res.

[57] Yeung PK (2018). Metabolomics and Biomarkers for Drug Discovery. Metabolites.

[58] Andrisic L, Dudzik D, Barbas C (2018). Short overview on metabolomics approach to study pathophysiology of oxidative stress in cancer. Redox Biol.

[59] Ussher JR, Elmariah S, Gerszten RE (2016). The Emerging Role of Metabolomics in the Diagnosis and Prognosis of Cardiovascular Disease. J Am Coll Cardiol.

[60] Johnson CH, Ivanisevic J, Benton HP (2015). Bioinformatics: the next frontier of metabolomics. Anal Chem.

[61] Beger RD, Dunn W, Schmidt MA (2016). Metabolomics enables precision medicine: "A White Paper, Community Perspective". Metabolomics.

[62] Puchades-Carrasco L, Pineda-Lucena A (2017). Metabolomics Applications in Precision Medicine: An Oncological Perspective. Curr Top Med Chem.

[63] Brunicardi FC, Gibbs RA, Wheeler DA (2011). Overview of the development of personalized genomic medicine and surgery. World J Surg.

[64] Arjmand B, Goodarzi P, Mohamadi-Jahani F (2017). Personalized Regenerative Medicine. Acta medica Iranica.

[65] Arjmand B, Abdollahi M, Larijani B (2017). Study Break: Precision Medicine: A New Revolution in Healthcare System. Iran Biomed J.

[66] Arjmand B, Larijani B (2017). Personalized Medicine: A New Era in Endocrinology. Acta Med Iran.

[67] Kottgen A, Raffler J, Sekula P (2018). Genome-Wide Association Studies of Metabolite Concentrations (mGWAS): Relevance for Nephrology. Semin Nephrol.

[68] Adamski J (2012). Genome-wide association studies with metabolomics. Genome Med.

